# Study protocol for a randomized controlled trial of a culturally adapted cardiovascular dance intervention in Mapuche women with obesity

**DOI:** 10.3389/fpubh.2026.1806558

**Published:** 2026-04-23

**Authors:** Cristóbal Riquelme-Hernández, Juan Pablo Reyes-Barría, Carlos S. Mancilla, Iván Cavero-Redondo, Edgar Vásquez-Carrasco, Paulina Sepúlveda, Cristian Sandoval-Vásquez, Igor Cigarroa

**Affiliations:** 1 Escuela de Kinesiología, Departamento de Salud, Universidad Arturo Prat, Victoria, Chile; 2 Kinesiología, Biocenter Fitness, Puerto Montt, Chile; 3 CarVasCare Research Group, Faculty of Nursing, University of Castilla-La Mancha, Cuenca, Spain; 4 School of Occupational Therapy, Faculty of Psychology, Universidad de Talca, Talca, Chile; 5 Centro de Investigación en Ciencias Cognitivas, Faculty of Psychology, Universidad de Talca, Chile; 6 VITALIS Longevity Center, Universidad de Talca, Talca, Chile; 7 Departamento de Ciencias Preclínicas, Facultad de Medicina, Universidad de La Frontera, Temuco, Chile; 8 Carrera de Terapia Ocupacional, Facultad de Ciencias de la Salud, Universidad Autónoma de Chile, Temuco, Chile; 9 Facultad de Medicina, Departamento de Medicina Interna, Universidad de La Frontera, Temuco, Chile; 10 Escuela de Kinesiología, Facultad de Ciencias de la Salud, Universidad Católica Silva Henríquez, Santiago, Chile

**Keywords:** cardiorespiratory fitness, cardiovascular diseases, dancing, exercise, obesity, quality of life

## Abstract

**Background:**

In rural Indigenous women, physical inactivity is a major modifiable risk factor for cardiometabolic illness and obesity. Thus, the objective was to describe the methodological design required to develop a randomized controlled trial to compare a culturally adapted cardiovascular dance intervention to traditional exercise on physical health, quality of life, and exercise-related psychosocial outcomes in rural Indigenous women with overweight/obesity as key determinants cardiometabolic conditions.

**Methods:**

Randomized, double-blind, parallel-group clinical trial. Twenty-two adults Mapuche women with overweight or obesity and cardiometabolic disorders from an Indigenous Community in Padre Las Casas, Chile, will be randomly allocated to a control group (*n* = 11) or a culturally adapted cardiovascular dance intervention group (*n* = 11). Two groups will receive one-hour supervision three times a week for 12 weeks. The primary goal is cardiorespiratory fitness, whereas secondary outcomes include physical health indices, health-related quality of life, motivation for physical activity, and perceived exercise barriers and advantages.

**Results:**

The culturally customized intervention is anticipated to yield increased levels of emotional well-being, perceived self-efficacy, and adherence in comparison to conventional exercise. The incorporation of Mapuche cultural elements, particularly traditional dancing, has the potential to enhance physical health, foster social engagement, reinforce cultural identity, and enhance community engagement.

**Conclusion:**

The objective of this trial is to evaluate the potential benefits and viability of culturally tailored physical activity interventions for Indigenous women. This will enhance the effectiveness of health promotion strategies that are context-specific and inclusive, as well as resolve a significant gap in the scientific literature.

**Clinical trials registration:**

Clinicaltrials.gov/study/NCT06903455, identifier NCT06903455

## 1 Introduction

Physical inactivity is a well-established modifiable risk factor for obesity, cardiovascular disease, and related cardiometabolic conditions (1, 2). In Chile, obesity prevalence among women is among the highest in Latin America, a region disproportionately affected by the global obesity epidemic (3). National surveillance data indicate that the prevalence of excess malnutrition among women reaches 78.7%, according to the National Health Survey (ENS). Moreover, evidence indicates that Indigenous women exhibit higher rates of overweight and obesity than non-Indigenous women, particularly between 20 and 49 years of age (4). Likewise, women report significantly lower levels of moderate-to-vigorous physical activity compared with men (5). These disparities are further exacerbated among lower socioeconomic groups, who present higher levels of sedentary behavior and greater structural barriers to engaging in regular physical activity (6, 7).

In the Chilean context, Mapuche individuals face a distinct set of barriers to engaging in physical activity. Qualitative studies conducted with Mapuche adults and Indigenous health program workers have documented challenges such as discrimination within health services, limited cultural relevance of physical activity programs, and gaps between mainstream health initiatives and Indigenous worldviews. These issues, together with insufficient professional training to deliver culturally adapted exercise strategies, contribute to reduced participation in structured physical activity among Mapuche communities. Additionally, cultural and socioeconomic marginalization disproportionately affects Mapuche women, who often encounter systemic barriers and reduced access to opportunities for physical activity, reinforcing the need for interventions that acknowledge cultural identity and community context (8).

Obesity is frequently accompanied by stigma, with women experiencing a disproportionate burden of weight-related disadvantage in domains critical to personal well-being (9). Cultural factors can reduce engagement in physical activity. Evidence from Hispanic adolescents with type 2 diabetes and Latino adults with type 2 diabetes shows that culturally rooted barriers (and related distress) are linked to lower adherence to exercise recommendations (10). As countries progress economically, the burden of overweight increasingly shifts toward socioeconomically disadvantaged groups, with an inverse association between socioeconomic status and body mass index observed in women but not in men (11). In Chile, poverty remains more prevalent in rural areas (13.82%) than in urban settings (10.42%). Within the Araucanía region, 2 municipalities are predominantly rural or mixed, including Padre Las Casas, which is classified as mainly mixed (12). According to the 2017 Population Census, this municipality has 76,126 inhabitants, of whom 40.35% reside in rural areas and 48.2% identify as Indigenous (Mapuche) (13).

Regular physical activity is an effective long-term strategy for managing overweight and obesity, resulting in reduced cardiovascular disease risk and improved cardiorespiratory fitness (14). Cardiovascular dance (CD) is a popular physical activity among women, associated with increased intrinsic motivation, reduced body weight and fat mass, and metabolic enhancements in individuals with overweight, obesity, and type 2 diabetes (15). Exercise adherence is notably influenced by program characteristics such as enjoyment, supervision, social support, cultural relevance, and adaptability to daily routines, in addition to support from professionals and peers (16, 17).

From a psychosocial perspective, self-determination theory (SDT) offers a robust framework for understanding motivation for physical activity (18). SDT posits that sustained engagement is driven by satisfaction of the basic psychological needs for autonomy, competence, and relatedness (19). Culturally relevant interventions may foster autonomous motivation and reduce perceived barriers, particularly among women experiencing stigma, low self-efficacy, or cultural tensions related to exercise participation (20).

Cardiovascular dance modalities -i.e., dance-based, music-driven aerobic exercise- such as Zumba have demonstrated high adherence and beneficial effects on weight maintenance and cardiorespiratory fitness (21). “Fitfolk,” a culturally grounded CD discipline originating in northern Chile, integrates fitness exercises with Chilean and Latin American folk music and dance, aiming to promote physical and psycho-emotional well-being while strengthening cultural identity (22). However, no studies in Chile or Latin America have evaluated Fitfolk or similar culturally adapted CD interventions as strategies to address overweight, obesity, or cardiometabolic disease, particularly among Indigenous women. To date, no randomized controlled trials have examined such interventions in rural Mapuche communities.

The comparator for this trial will be a conventional physical exercise program, defined according to current clinical guidelines and standard care practices (23, 24), and aligned with recommendations from the European Association for the Study of Obesity (25). Both intervention and control groups will receive supervised exercise three times per week for 60 min over 12 weeks, ensuring equivalent exercise dose and professional contact time, thereby allowing isolation of the cultural component as the primary differentiating factor.

## 2 Research question

Is a culturally adapted cardiovascular dance program (Fitfolk) as effective as, or more effective than, a conventional physical exercise program in improving physical health, health-related quality of life, and exercise motivation, and in reducing perceived barriers to physical activity among rural Indigenous women with overweight or obesity and cardiometabolic risk in the commune of Padre Las Casas, Chile?

## 3 Study objective

To describe the methodological design of a randomized controlled trial aimed at evaluating the effects of a culturally adapted cardiovascular dance program (Fitfolk), compared with a conventional physical exercise program, on physical health outcomes, health-related quality of life, exercise motivation, and perceived barriers to physical activity in rural Indigenous women with overweight or obesity as key determinants cardiometabolic diseases from the commune of Padre Las Casas, Chile.

## 4 Methods

### 4.1 Design

This research will be structured as a randomized controlled trial (RCT) featuring two parallel arms, with blinding implemented for both the outcome assessor and the statistical analyst. The trial followed CONSORT guidelines (26) and complied with the updated SPIRIT guidelines (27) for RCTs, with the SPIRIT checklist (Supplementary Table 1). The TIDieR checklist was utilized to facilitate clear and reproducible reporting of the clinical intervention (28).

The Scientific Ethics Committee of Universidad Santo Tomás conducted a review and granted approval for the study protocol, in compliance with the ethical requirements for clinical studies (Approval code No. 231366443/2023). The RCT protocol was prospectively registered in December 2024 in the ClinicalTrials.gov database (Registration Number NCT06903455), and the comprehensive statistical analysis plan is available to the public.

### 4.2 Study setting

A formal agreement was established with the Huichacura-Cayuqueo Indigenous Community of Padre Las Casas to implement the FONDEPORTE project (Project N° 2,400,120,062). This Mapuche community comprises approximately 85 families, totaling around 170 members, and is recognized as one of the largest Indigenous communities in the rural sector of the La Araucanía Region. The community’s daily practices reflect traditional Mapuche cultural patterns, which are evident in their belief systems, customary dietary practices, and habitual physical activities.

Within the context of the study, women from the community who met the predefined eligibility criteria will be identified and invited to participate through informational meetings and digital outreach strategies.

### 4.3 Sample size

**Figure 1:**
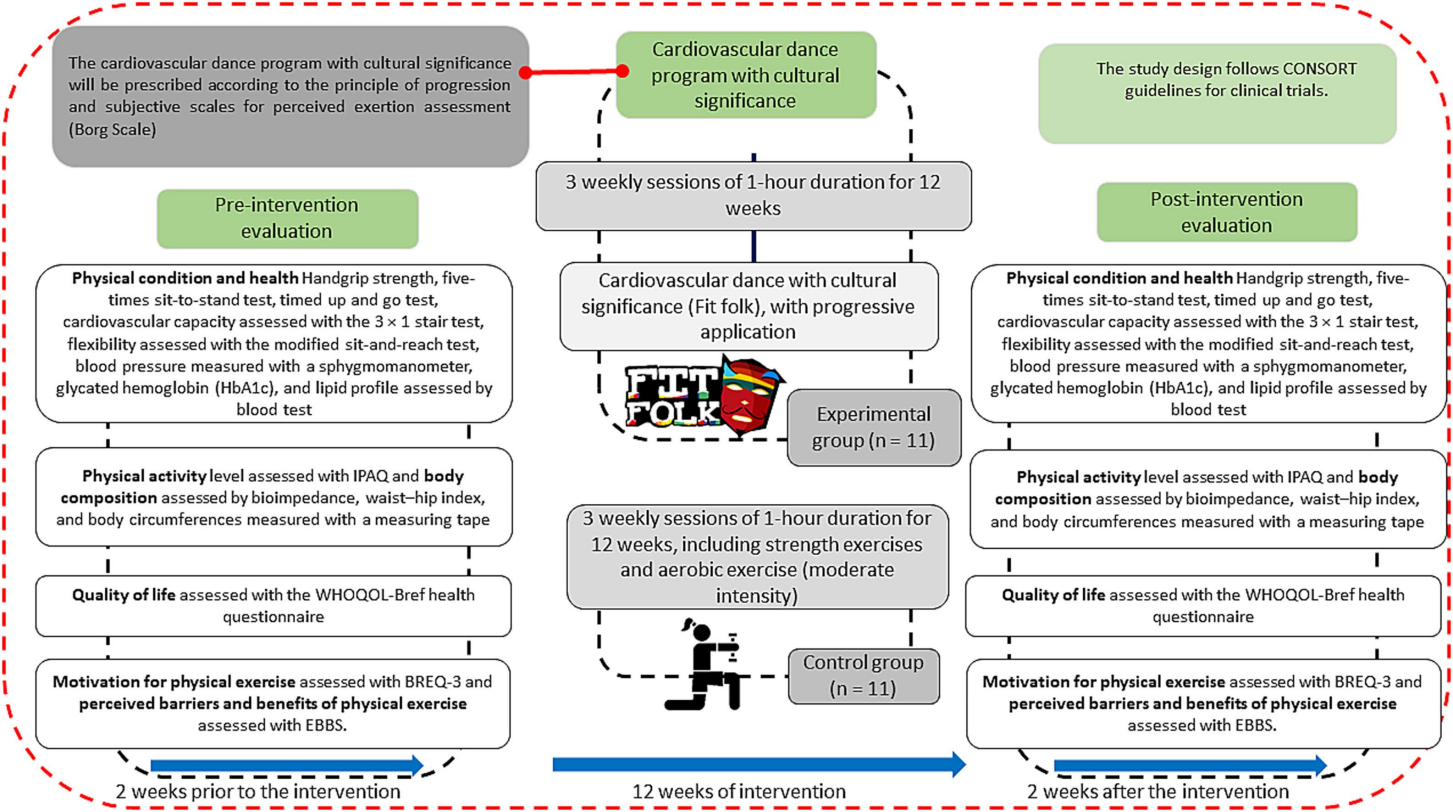
Flow chart for participant inclusion and follow-up: selection, inclusion, exclusion, and randomization (98).

The sample size was determined *a priori* using G*Power software (version 3.1.9.7, Heinrich Heine University Düsseldorf, Düsseldorf, Germany) for a pre–post intervention comparison of mean differences. Based on an anticipated effect size of 0.8 for aerobic capacity, derived from previously published data (29), a minimum of 16 participants (eight per group) was required to achieve a statistical power of 80% (1 − *β* = 0.80) with a two-sided significance level of *α* = 0.05. To account for potential attrition, an additional six participants will be recruited, resulting in a total sample of 22 individuals (11 per group; Figure 1).

### 4.4 Participant recruitment

An open invitation will be extended to all adult women from the Huichacura-Cayuqueo Indigenous Community in the municipality of Padre Las Casas who meet the established eligibility criteria. Participant recruitment will be conducted through in-person informational meetings held at the community headquarters, complemented by digital dissemination coordinated in collaboration with the community leadership. Eligible participants will include sedentary adult Mapuche women (30, 31) with overweight or obesity and medically controlled cardiometabolic conditions, as verified through documented medical diagnoses, routine clinical follow-ups, and prescribed treatments provided by their respective primary health care centers. Participants must also demonstrate the physical ability to safely engage in moderate-intensity group exercise without assistance and have sufficient availability to attend the scheduled intervention sessions.

Individuals with medical conditions that contraindicate participation in group physical activity, including neurological events such as stroke or acute and chronic respiratory diseases, will be excluded. Detailed inclusion and exclusion criteria are presented in Table 1.

**Table 1:** Inclusion and exclusion criteria.

Inclusion criteria	Exclusion criteria
Be a woman aged 18 to 65 years.	To have organic pathologies that prevent physical activity, such as stroke, acute or chronic respiratory diseases.
Having a high level of sedentary lifestyle (more than 4 h per day) (30).	To have suffered a recent acute myocardial infarction (in the last 3 months).
To have a diagnosis of malnutrition due to overweight or obesity (based on BMI).	To have a cardiovascular pathology that prevents group physical activity.
If suffering from chronic diseases diagnosed by a doctor, such as diabetes, hypertension, or dyslipidemia, these must be properly controlled (medication use and corresponding medical checkups). with stable treatment for ≥3 months, including no recent changes in medication, no acute exacerbations, and ongoing clinical follow-up.	Having uncontrolled hypertension or diabetes mellitus, defined as lacking medical or pharmacological treatment, lacking clinical follow-up, or presenting clinical values that contraindicate participation in group exercise.
Be willing to exercise and have time available between July and September 2024 (3 sessions per week, one hour each).	Neurological, respiratory, or systemic diseases that impair the ability to perform exercise (e.g., stroke, severe chronic respiratory disease).
To have time available for an evaluation before the intervention program in July and another at the end of the program in October (each lasting approximately 2 h).	Participate in another physical exercise program during the duration of the project.
Belong to the Huichacura Cayuqueo Indigenous Community.	Use of active obesity treatments, including pharmacological therapy for weight loss (e.g., naltrexone/bupropion or agents associated with significant gastrointestinal or exercise-limiting side effects) or bariatric surgery.
Demonstrate the ability to safely perform moderate-intensity exercise, confirmed through baseline clinical screening and a submaximal 3 × 1-min step test without adverse symptoms.	Not want to participate and not sign the consent form.
Be responsible with the schedules set for the evaluation and intervention.	

### 4.5 Randomization

Eligible participants will be randomly allocated to study groups following completion of baseline assessments. The randomization sequence will be generated using computer-based random number software by a co-investigator who is independent of participant recruitment and assessment procedures. Stratified randomization based on age and body weight will be applied, using permuted blocks of variable size to ensure balanced group allocation.

Allocation concealment will be maintained through the use of sequentially numbered, opaque, sealed envelopes prepared in advance by the co-investigator responsible for the randomization sequence. Envelopes will be opened only after participants have completed all baseline evaluations. Participants will then be assigned to one of two study arms: “G1” the Fit-Folk Cardiovascular Dance (FCD) group, which will receive the culturally adapted dance-based intervention, or “G2” the Conventional Physical Exercise (CPE) group, which will follow a standard exercise program.

Outcome assessors will remain blinded to group allocation. All evaluations will be conducted by personnel not involved in intervention delivery, following standardized scripts, and in separate facilities and schedules to prevent contact with intervention activities. Participants will be instructed not to disclose their group assignment during assessments. The statistical analyst will also remain blinded: an independent investigator will recode the dataset into ‘Group A’ and ‘Group B’ prior to analysis, and unblinding will occur only after completion of the primary analyses. Due to the behavioral nature of the interventions, participants cannot be blinded to their assigned program; however, this does not compromise the blinding of assessors or analysts.

### 4.6 Procedure

The full study cohort will undergo in-person evaluations to assess primary and secondary outcomes two weeks before the intervention begins. Assessment appointments will be arranged through text message and subsequently verified by telephone. At the baseline visit, written informed consent will be secured, participants will be provided with hydration bottles, and a unique identification code will be assigned and documented.

**Figure 2:**
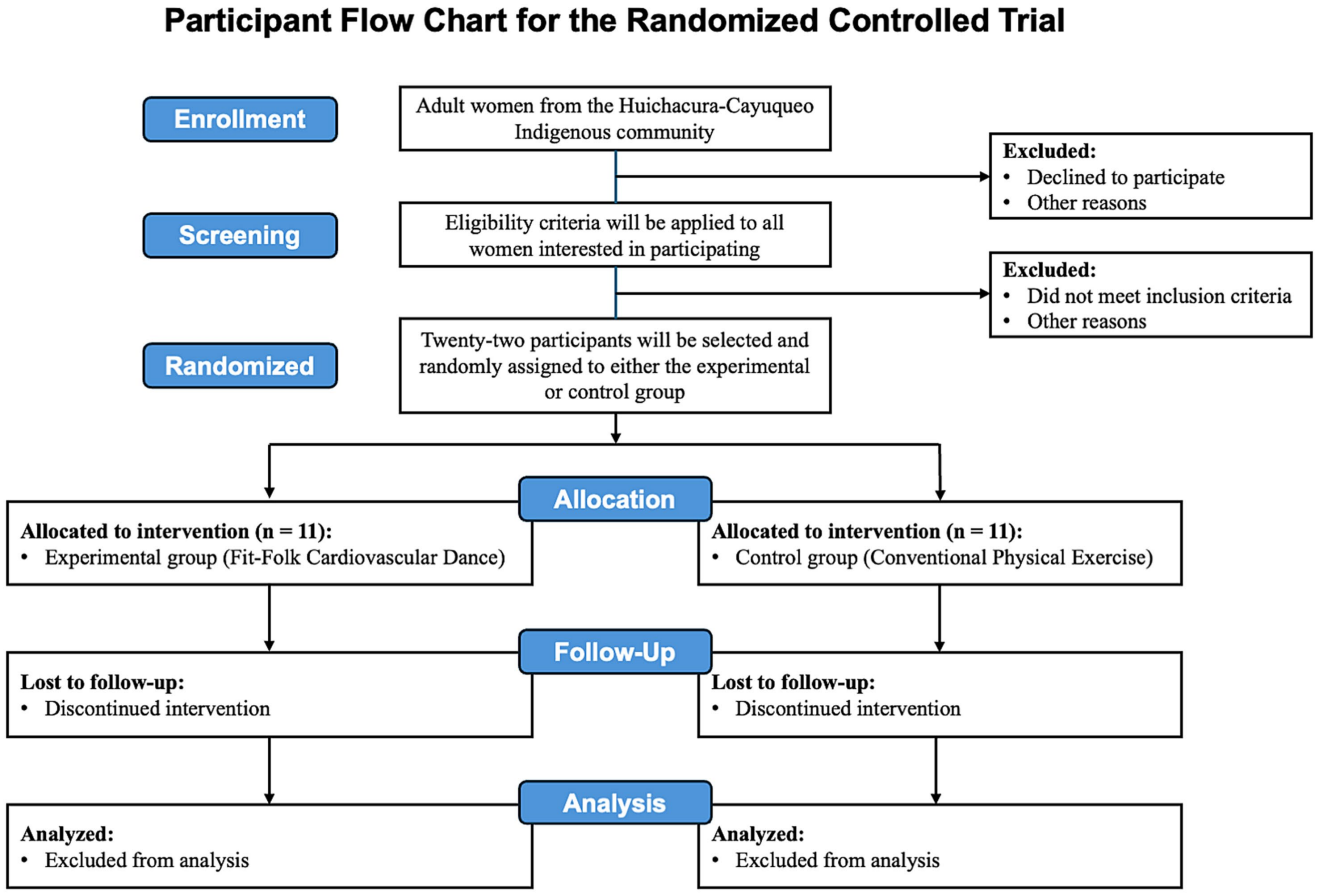
Study design of the intervention program based on culturally relevant cardiovascular dance. BMI, Body mass index; BREQ-3: Behavioral Regulation in Exercise Questionnaire-3; CONSORT: Consolidated Standards of Reporting Trials; EBS, Exercise Benefits Scale; FPT-folk: Functional progressive training with cultural significance; IPAQ-SF, International Physical Activity Questionnaire – Short Form.

During the intervention period, participant attendance will be systematically recorded, and a digital registry will be employed to document covariates that may affect outcome measures. At the conclusion of the 12-week intervention, all study variables will be re-evaluated utilizing the identical procedures (Figure 2).

All members of the technical team will participate in standardized training to guarantee uniform and impartial execution of assessment protocols. Evaluations will take place in a specially designed facility that ensures participant privacy, thermal comfort, and adequate control of lighting and ventilation. Assessments will be scheduled at consistent times of day for each participant and under conditions similar to baseline whenever possible. Participants will be directed to avoid vigorous physical activity for 24 h before the assessment, adhere to their usual diet, and ensure they are sufficiently rested. Measurements will be conducted by certified health professionals. To reduce assessment bias, outcome evaluators will maintain independence from the team implementing the intervention.

### 4.7 Adverse event and safety monitoring

The intervention, which includes dance-based activities and supervised physical exercise, is categorized as minimal risk. A systematic framework will be established to monitor and document adverse events rigorously. Instructors will document instances of discomfort, minor injuries, cardiovascular symptoms, exercise intolerance, or other unforeseen events during each intervention session. All adverse events will be classified by severity and evaluated for potential correlation with the intervention, subsequently reported to the lead investigator.

Participants experiencing an adverse event will be referred to the appropriate health care facility (Servicio Alta Resolución Conunhuenu) for clinical assessment as required. Decisions about ongoing participation in the study will be determined individually, prioritizing participant safety.

To further strengthen safety procedures and in alignment with the Exercise is Medicine framework, exercise prescription will be initiated only after confirming each participant’s clinical stability and functional capacity. Training intensity and progression will be individualized according to participants’ health status and medication profiles, and instructors will continuously monitor physiological responses during sessions to minimize potential drug–exercise interactions and ensure appropriate and safe adaptation to the training program.

### 4.8 Intervention

The design and reporting of the experimental and control interventions were aligned with the TIDieR guidelines to ensure transparency and reproducibility of clinical procedures (28).

### 4.9 Fit-folk cardiovascular dance initiative

#### 4.9.1 Title of the intervention and its justification

The experimental intervention involved a group-based Fit-Folk Cardiovascular Dance program based on Chilean and Latin American folk dances. This approach was chosen due to evidence indicating that cardiovascular dance modalities positively impact cardiovascular health (32), increase maximal oxygen uptake (VO₂max) (21), and enhance health-related quality of life (33).

#### 4.9.2 Materials and setting

The intervention will require minimal equipment, including a digital blood pressure monitor and an amplified sound system. Musical selections and choreographies will be derived from Chilean and Latin American folk traditions. Sessions will be conducted at a community venue within the rural setting, offering sufficient space, a safe floor surface suitable for dynamic movement, and optional exercise mats for post-session stretching. Communication between participants and instructors will be facilitated through digital messaging platforms. No additional resistance equipment or specialized sportswear will be required.

##### 4.9.2.1 Procedures

At the start of each session, participants’ blood pressure and heart rate will be measured to ensure safe participation in physical activity. Each session followed a standardized structure (Table 2). Although the intervention targets Mapuche women, traditional Mapuche ceremonial dances will not be incorporated due to their spiritual and ritual significance. Instead, Fit-Folk’s cultural adaptation is reflected in: (a) the use of folk and popular dance styles widely practiced in rural southern Chile, such as the cueca, rancheras, and cumbia, which are familiar and culturally significant to community members; and (b) the incorporation of deeply structured cultural elements, such as coordination with the Huichacura-Cayuqueo Indigenous Community, an all-female group format, culturally safe communication, and community-agreed norms. This approach ensures cultural relevance while maintaining respect for Mapuche ceremonial traditions.

**Table 2:** Structure of a Fit-Folk cardiovascular dance class.

Phase	Duration	Activity description	Intensity (Borg)	Dance styles	Country/folklore
Warm-up	8–10 min (2 to 3 songs)	Slow and progressive movements including joint mobility, basic dance steps, and dynamic stretching, aimed at increasing joint range of motion, body temperature, and cardiorespiratory activation.	Moderate, Borg 3–4	Huayno; Nicaraguan full-rhythm dances	CL and PE
Main development	35–40 min (14 to 16 songs)	The main phase of the session, characterized by higher-intensity rhythms and movements. Participants replicate Latin American folk choreographies guided by the instructor, emphasizing continuous movement and aerobic demand.	Moderate to Intense, Borg 5–9	Moderate-intensity dances: Tumbe; Afro-descendant Chilean rhythms from Arica; Colombian cumbia; Southern Chilean rancheras; Afrosaya (Bolivia); Chilean cueca. High-intensity dances: Tinkus; Forró (Brazil); Caporal; El Rin (Chile); Merengue.	BO, BR, CL, CO and PA
Cool-down/return to calm	8–10 min (2 to 3 songs)	Gradual reduction of movement intensity using slower dance steps, followed by static stretching to promote relaxation and recovery.	Low, Borg 1–3	Morenada; Venezuelan gaita; Peruvian waltz.	BO PE and VE

Borg, Borg rating of perceived exertion; BO, Bolivia; BR, Brazil; CL, Chile; CO, Colombia; PA, Panama; PE, Peru; VE, Venezuela.

##### 4.9.2.2 Provider

Sessions will be conducted by a certified Fit-Folk cardiovascular dance instructor who has prior experience in implementing community-based interventions in rural regions of Padre Las Casas. The instructor will be compensated financially for professional services rendered.

##### 4.9.2.3 Format and location

Intervention sessions will be conducted in person and in a group format, consisting of 11 participants per group, at the Huichacura-Cayuqueo community center, situated in the rural area where the participants reside.

##### 4.9.2.4 Frequency, duration, and progression are critical parameters in the analysis of various phenomena

Sessions occurred three times weekly (Monday, Wednesday, and Friday), lasting approximately 60 min each, throughout a 12-week intervention period. In the first three weeks, sessions focused on the introduction of dance steps and choreography. During this phase, cardiovascular training utilized an intermittent structure, alternating between demonstration and practice intervals, with exercise intensity sustained at a moderate level (3–4 on the modified Borg scale) (34, 35).

Training demands subsequently increased progressively through adjustments in musical tempo, choreography complexity, and movement intensity, leading to vigorous-to-high intensity exertion (modified Borg scale: 5–9) (35). This progression adhered to the principle of progressive overload (36), consistent with previous cardiovascular dance interventions (15, 37), and was articulated using the FITT framework as recommended by the American College of Sports Medicine (38).

##### 4.9.2.5 Adaptations and safety considerations

Standardized adaptation strategies will be applied consistently across both the experimental and control groups. Sessions will be modified or temporarily suspended on an individual basis when altered physiological parameters will be detected. Participants presenting resting blood pressure values >180/110 mmHg at session onset will be advised to regulate blood pressure pharmacologically and to perform exercise at moderate intensity. Participation will be suspended if systolic blood pressure exceeded 220 mmHg and/or diastolic blood pressure exceeded 105 mmHg, or in cases of hypotension during the session (38, 39).

For participants with diabetes, exercise will be avoided if pre-session blood glucose levels will be <90 mg/dL or >250 mg/dL (40). These glucose thresholds will be based on participants’ routine home self-monitoring of capillary blood glucose, as recommended in standard diabetes care. At the beginning of each training day, participants will be asked to report whether their most recent home reading falls within a safe range for exercise. On-site screening will include only blood pressure and heart rate measurements.

##### 4.9.2.6 Modifications and adherence

Any protocol modifications will be documented in the intervention log and reported in the final manuscript. Attendance at each session will be recorded, and constant communication will be maintained between participants and instructors through a dedicated WhatsApp group.

##### 4.9.2.7 Intervention fidelity

Session fidelity will be assessed using post-session satisfaction surveys. Additionally, digital questionnaires will be administered throughout the intervention to capture covariates related to behavioral changes and barriers associated with participation in Fit-Folk Cardiovascular Dance activities.

### 4.10 Conventional physical exercise program

#### 4.10.1 Title of the intervention and its justification

The control condition will consist of a group-based conventional physical exercise program combining resistance and cardiorespiratory training. This intervention was selected due to its documented effectiveness in improving anthropometric, metabolic, and cardiorespiratory outcomes (25, 41).

#### 4.10.2 Materials and setting

Equipment included kettlebells (4, 6, and 8 kg), dumbbells (2 and 3 kg), suspension trainers, elastic resistance bands, medicine balls (3 and 5 kg), exercise mats, agility ladders, cones, amplification equipment, and supportive community infrastructure.

#### 4.10.3 Procedures

Each session will be organized into structured blocks. The initial warm-up block included dynamic stretching (42), joint mobility exercises, movement-specific preparatory drills (43), and neuromuscular activation.

The primary training block will emphasize high neuromuscular demand exercises (e.g., squats, deadlifts, overhead presses, rows), with exercise selection based on planned movement patterns and load prescription tailored to training objectives. Resistance training intensity will be monitored using the OMNI-RES perceived exertion scale (44).

Next, a secondary resistance block will be performed, characterized by reduced intensity and volume. An auxiliary block will be then implemented, focusing on core strengthening (45), joint mobility, large-joint stability, and advanced technical movements.

The metabolic conditioning block incorporated interval-based methods targeting both cardiorespiratory and muscular systems, monitored using the speech test (46) and Borg scale (47), including high-intensity interval training (HIIT) protocols when appropriate (48). Sessions concluded with a cool-down phase consisting of breathing exercises, joint mobilization, and static stretching (49, 50).

#### 4.10.4 Provider

Sessions will be delivered in person by a qualified physical education professional with experience in adult fitness training.

#### 4.10.5 Format and location

Group size will be consistent with the experimental condition (11 participants per group), and all sessions will be held at the Huichacura-Cayuqueo community center.

#### 4.10.6 Training structure and frequency

A full-body training approach (51) using mixed training methods (52) will be employed. The 12-week program will be divided into three mesocycles: anatomical adaptation/general strength (weeks 1–4), hypertrophy/general strength (weeks 5–8), and muscular endurance/general strength (weeks 9–12). Exercise selection and distribution will be based on fundamental movement patterns and upper- and lower-limb push–pull dynamics (53). Training frequency and session duration matched the experimental group (three sessions per week, ~60 min each) (54).

### 4.11 Intervention duration

The intervention will be conducted over a three-month period. Figure 1 presents a flow diagram outlining participant inclusion, follow-up procedures, and the timing of baseline and post-intervention assessments of the study variables.

### 4.12 Participant compliance and follow-up criteria

Participant adherence will be defined as attendance at ≥80% of the scheduled FCD or CPE sessions (55). Attendance will be documented at the start of each session using a standardized recording form. Primary analyses will follow the intention-to-treat principle. In addition, a per-protocol sensitivity analysis will be performed, restricted to participants who attended at least 80% of the intervention sessions.

### 4.13 Analysis variables

In both study arms, assessments will be conducted at baseline and post-intervention to evaluate physical health parameters, health-related quality of life, motivation for physical activity, and perceived barriers to and benefits of exercise. Data will be collected using standardized case report forms and subsequently entered into a secure database incorporating quality control procedures, including range checks and automated alerts for missing or inconsistent values. All assessments will be performed by qualified health professionals who will remain blinded to group allocation. Prior to study initiation, outcome assessors will receive structured training to standardize measurement procedures and ensure consistent application of assessment protocols, thereby enhancing reliability and minimizing measurement bias (56).

All assessment activities will take place at the Huichacura-Cayuqueo community center, following a scheduled appointment system and in a facility designed to ensure participant privacy and appropriate environmental conditions, including controlled temperature and humidity. Blood samples will be collected and analyzed at an accredited laboratory within the municipality of Padre Las Casas, in accordance with institutional procedures. Participants will be instructed to observe a fasting period of at least 8 h and no more than 12 h prior to blood collection to ensure analytical accuracy.

### 4.14 Main variables

#### 4.14.1 Cardiorespiratory capacity

Cardiorespiratory fitness will be the primary outcome of the trial and will be assessed using the 3 × 1 Step Test (ST3 × 1), a validated protocol for estimating VO₂max in adults with cardiovascular risk factors (57). During the test, participants will repeatedly step up and down from a standardized platform (0.20 m in height) across three progressively demanding 1-min stages, each separated by a 1-min recovery period. Heart rate and blood pressure will be measured immediately upon test completion and again after one minute of recovery.

Test-derived estimates will be recorded and interpreted using age- and sex-specific normative reference values. Results falling below the established percentile thresholds will be classified as indicative of diminished cardiorespiratory fitness (58).

To ensure participant safety during the 3 × 1 Step Test, trained evaluators will monitor blood pressure and heart rate before the test, conduct continuous observation for signs of exercise intolerance during the protocol, and repeat cardiovascular measurements immediately after completion and during the recovery period. The test will be discontinued if any adverse symptoms occur, and participants exhibiting abnormal responses will be referred for clinical evaluation.

#### 4.14.2 Strength

##### 4.14.2.1 Upper extremity strength

Handgrip strength will be assessed using a calibrated hand dynamometer, which is a recognized metric for overall muscular strength and physical function in adult populations (59, 60). The evaluation will adhere to the standardized protocol established by the American Society of Hand Therapists (ASHT) (61). Participants will be positioned with the shoulder in a neutral alignment, the elbow flexed at 90 degrees, the forearm in a neutral orientation, and the wrist set between 0 and 30 degrees of extension. A maximum of three attempts will be conducted with each hand in an alternating sequence, incorporating rest intervals of no less than 30 s between trials. The maximum recorded value (kg) will be utilized for further analyses. Results will be analyzed utilizing age- and sex-specific normative reference values pertinent to the Chilean population (62).

##### 4.14.2.2 Lower limb strength

The five-times sit-to-stand test (5 × STS) will be utilized to assess lower limb muscle strength, as it is a validated and commonly employed functional measure of lower extremity strength (63). The assessment measures the duration (in seconds) taken by participants to stand up from and sit back down in a standard chair (height 43–45 cm) for five consecutive repetitions, with arms crossed over the chest to eliminate assistance (64).

### 4.15 Secondary variables

#### 4.15.1 Lower limb functionality

Lower limb functional performance will be assessed using the Timed Up and Go (TUG) test, a well-established measure of functional mobility and dynamic postural control in adult populations (65, 66). The test records the time (in seconds) required for participants to rise from a seated position, walk three meters at a comfortable pace, turn, return to the chair, and sit down. The TUG demonstrates excellent test–retest reliability (intraclass correlation coefficient >0.95) and strong concurrent validity with other indicators of mobility and functional balance across different age groups (67). Longer completion times reflect poorer functional mobility and dynamic balance. Results will be interpreted using age- and sex-specific normative reference values for adults (68).

#### 4.15.2 Lower limb flexibility

Flexibility of the lower limbs will be evaluated using the modified seated reach test, which estimates extensibility of the posterior muscular chain, particularly the hamstrings and lumbar region, in adults. Participants will sit on a standard chair with one foot flat on the floor and the contralateral leg extended forward, heel in contact with the ground. With the extended knee maintained in full extension, participants will slowly lean forward and reach as far as possible with both arms. Reach distance will be measured in centimeters from a fixed reference point at the toes (69). One familiarization trial will be performed, followed by two valid attempts, with the best value retained for analysis.

#### 4.15.3 Blood pressure

Resting blood pressure will be measured using a validated automatic digital sphygmomanometer for the assessment of systolic and diastolic blood pressure in adults (70). Measurements will be obtained with participants seated, the arm supported at heart level, and after a minimum of five minutes of rest, in accordance with American Heart Association guidelines for standardized blood pressure assessment (71). Two measurements will be recorded, one immediately before the training session and one at its conclusion, using identical procedures for both study groups.

#### 4.15.4 Metabolic health

Metabolic health will be evaluated through the assessment of glycosylated hemoglobin (HbA1c), lipid profile parameters, and other routinely used biochemical markers indicative of glycemic control and cardiovascular risk in adults (72, 73).

Venous blood samples will be collected by certified phlebotomists at an accredited clinical laboratory following standardized pre-analytical procedures. All samples will be obtained in the morning after an overnight fasting period of 8–12 h and processed according to established laboratory protocols. Analyses will be performed using validated automated systems, with internal quality control procedures ensuring analytical accuracy and reproducibility.

HbA1c will be measured from venous blood samples using methods standardized by the National Glycohemoglobin Standardization Program (NGSP) and traceable to the Diabetes Control and Complications Trial (DCCT). Results will be expressed as percentages (%). HbA1c values ≥5.7% will be interpreted as impaired glucose regulation, while values ≥6.5% will be considered diagnostic of diabetes mellitus, in accordance with American Diabetes Association (ADA) criteria (74).

The lipid profile will include total cholesterol, high-density lipoprotein (HDL) cholesterol, low-density lipoprotein (LDL) cholesterol, and triglycerides, measured using enzymatic colorimetric methods. LDL cholesterol will be calculated or directly measured according to laboratory standards. Results will be interpreted in accordance with the clinical recommendations of the European Society of Cardiology and the European Atherosclerosis Society (73).

#### 4.15.5 Physical activity level

The International Physical Activity Questionnaire (IPAQ) short form will be employed to evaluate physical activity, providing a standardized method to measure the frequency, duration, and intensity of activities performed in the preceding seven days. The IPAQ demonstrates sufficient validity and reliability in assessing physical activity in adult populations within both clinical and community settings (75). Weekly energy expenditure will be measured and expressed in metabolic equivalent minutes per week (MET-min/week), calculated from the duration of vigorous-intensity activity, moderate-intensity activity, and walking. Participants will be categorized into low, moderate, or high physical activity levels according to the criteria established by the questionnaire developers (76).

#### 4.15.6 Body composition

Body composition will be assessed using bioelectrical impedance analysis with the InBody 270 device (InBody Co., Ltd., South Korea). This method quantifies fat mass, fat-free mass, and total body water through the analysis of body resistance and reactance via a low-intensity alternating electrical current (77).

#### 4.15.7 Quality of life

The World Health Organization Quality of Life–BREF questionnaire (WHOQOL-BREF) will be employed to evaluate individuals’ self-reported well-being across many aspects. This measure will be employed to assess health-related quality of life. The WHOQOL-BREF exhibits robust reliability and validity in adult populations across many sociocultural situations (78). The questionnaire consists of 26 items divided into four domains: physical health, psychological well-being, social relationships, and environmental context, in addition to two general items evaluating perceived health status and overall quality of life. Responses are evaluated using a five-point Likert scale, with higher scores signifying an improved subjective quality of life. Domain scores are normalized to a 0–100 scale to facilitate comparisons across domains and populations (79).

#### 4.15.8 Motivation for physical exercise

The Behavioral Regulation in Exercise Questionnaire–3 (BREQ-3) (80, 81) will be employed to evaluate motivation for physical exercise. This instrument is based on self-determination theory and is designed to capture various forms of motivational regulation that influence exercise adherence. In adult and physically active populations, the BREQ-3 has exhibited cross-cultural validity and sound psychometric properties (82). The questionnaire comprises 24 items that are organized into six subscales that represent varying degrees of self-determination: amotivation, external regulation, introjected regulation, identified regulation, integrated regulation, and intrinsic motivation. Scores are determined on a five-point Likert scale, with a score of 0 indicating “not at all true for me” and a score of 4 indicating “completely true for me.” Higher scores indicate a higher degree of autonomous motivation. The Relative Self-Determination Index (SDI) can be used to summarize outcomes or to analyze them at the subscale level, as it represents the motivational continuum described by the theory (83).

#### 4.15.9 Barriers and benefits of physical exercise

The Exercise Benefits/Barriers Scale (EBBS), a validated instrument, will be employed to evaluate the perceived advantages and obstacles of physical exercise, with a particular emphasis on the psychosocial factors that influence regular physical activity participation. The EBBS demonstrates robust psychometric qualities, including outstanding internal consistency and construct validity, across a variety of adult populations. The scale is composed of 43 items that are categorized into two dimensions: perceived benefits (29 items) and perceived barriers (14 items) that are associated with physical activity (84, 85).

#### 4.15.10 Sociodemographic variables and lifestyles

Participants’ sociodemographic characteristics, including age, residential area (urban or rural), schooling completed (primary: <8 years; secondary: 8–12 years; additionally higher education: >12 years), and the presence of chronic conditions such as hypertension, diabetes mellitus, and dyslipidemia, will be collected. Additionally, lifestyle behaviors, including tobacco consumption and alcohol consumption, will be recorded. Additionally, concise surveys will be implemented to assess sleep patterns and dietary habits. All questionnaires and functional assessment tools used in this protocol have demonstrated acceptable validity and reliability in Chilean adult populations.

### 4.16 Statistical analysis

Multiple imputation methods will be employed to address missing data in accordance with the intention-to-treat principle (86). Descriptive statistics for continuous variables will be presented using measures of central tendency and dispersion, whereas categorical variables will be represented through frequencies and percentages. The Shapiro–Wilk test assesses data distribution, whereas Levene’s test evaluates the homogeneity of variances.

Non-parametric sensitivity analyses will be conducted when the criteria for normality or homoscedasticity are not met, due to the limited sample size (*n* = 22) and potential violations of parametric assumptions. Wilcoxon signed-rank tests will be used for within-group comparisons, while Mann–Whitney U tests will be applied for between-group comparisons.

A two-way repeated-measures analysis of variance (ANOVA) will be conducted to assess intervention effects, incorporating group (experimental vs. control), time (pre-intervention vs. post-intervention), and the group × time interaction as model factors. Bonferroni-adjusted *post hoc* tests will be utilized where necessary to determine statistically significant differences. Effect sizes will be computed utilizing Cohen’s d and classified as inconsequential (<0.20), small (0.20–0.49), moderate (0.50–0.79), or large (≥0.80) (87). Statistical analyses will be performed utilizing SPSS software (version 25; IBM Corp., Chicago, IL, USA), with a significance threshold established at *p* < 0.05. Graphical analyses will be conducted with GraphPad Prism (version 8, GraphPad Software, San Diego, CA, USA).

### 4.17 Ethical implications and data management

The study received ethical approval from the Scientific Ethics Committee of Universidad Santo Tomás (Resolution No. 231366443/2023). The study protocol was registered on ClinicalTrials.gov. All participants provided written informed consent prior to the initiation of any study-related procedures. Participant data will be managed in accordance with strict confidentiality standards, using coded identifiers and secure data storage systems. Participation in the study is voluntary, and individuals may withdraw at any time without any impact on their usual health care or related services.

## 5 Discussion

This protocol aims to evaluate the effects of a culturally grounded cardiovascular dance intervention in women with obesity from a Mapuche community through a randomized controlled trial. The proposed approach integrates physical activity rooted in cultural identity as a strategy to promote metabolic health and strengthen community well-being.

Obesity represents a global public health challenge and is among the most important modifiable risk factors for chronic disease and premature mortality. Its prevalence has increased markedly over recent decades, with a reported rise of more than 100% among women since 1990, and projections indicating that over half of the adult population may be affected by obesity by 2050 (88). Evidence suggests that Indigenous populations experience obesity prevalence rates comparable to or exceeding those observed in non-Indigenous groups (89–91), highlighting persistent health inequities and the urgent need for culturally responsive prevention and treatment strategies.

Within this context, culturally meaningful cardiovascular dance emerges as a novel and promising alternative to conventional physical activity interventions. Experimental studies have demonstrated that dance-based exercise programs can produce favorable effects on adiposity and cardiometabolic risk markers (92–94). Moreover, interventions grounded in cultural practices have been associated with additional benefits, including enhanced cultural identity, increased community cohesion, and improved adherence to physical activity (94, 95). These dimensions are particularly relevant in rural Indigenous settings, where social belonging and cultural continuity play a central role in health-related behaviors. Incorporating culturally relevant elements into exercise interventions may further enhance self-efficacy, emotional well-being, and sustained engagement in physical activity (96, 97). In the Mapuche context, folk dance constitutes both a form of physical movement and a mode of cultural expression, offering an opportunity to simultaneously address physical health, identity affirmation, and collective participation.

Notably, even without statistically significant metabolic effects, the results of this study would offer important insights into the function and constraints of cultural elements in exercise-based therapies. These results may suggest that metabolic outcomes are more significantly affected by training volume and intensity than by cultural adaptation alone, or that extended intervention durations and greater community engagement are necessary to produce substantial physiological changes.

This protocol is characterized by its incorporation of stringent quantitative methods within an intercultural framework, targeting a population that has been traditionally marginalized in clinical research. The study enhances the acknowledgment of Indigenous views in health research by including Mapuche women and appreciating traditional knowledge in the intervention design. The findings may guide the formulation of intercultural health policies and community-based initiatives that integrate Indigenous identity as a fundamental element of preventive and treatment approaches.

Expected limitations encompass difficulties in maintaining long-term compliance, potential selection bias, and logistical obstacles associated with doing research in rural community environments. The study aims to produce evidence that endorses the creation of culturally customized intervention models for obesity management and enhances the cultural significance of physical and mental health promotion programs within Indigenous groups.

This protocol presents a novel, comprehensive, and culturally attuned method for addressing obesity in Mapuche women. This approach integrates scientific evidence with sociocultural context to produce transferable and socially relevant knowledge consistent with intercultural health principles, while offering a solid clinical framework to inform future interventions in Indigenous communities.
